# A pilot evaluation of scalp skin wounding to promote hair growth in female pattern hair loss

**DOI:** 10.1016/j.ijwd.2020.11.006

**Published:** 2020-12-08

**Authors:** Laura J. Burns, Dina Hagigeorges, Kelly E. Flanagan, James Pathoulas, Maryanne M. Senna

**Affiliations:** a Department of Dermatology, Massachusetts General Hospital, Boston, MA, United States; b Harvard Medical School, Boston, MA, United States

**Keywords:** Female pattern hair loss, FPHL, Microneedling, Scalp microwounding

Dear Editors,

Despite high population prevalence, effective treatments for female pattern hair loss (FPHL) remain limited (Van Zuuren et al., 2012). Options can be further restricted when emerging treatments are tested only in men because length, styling, density, and patterning of hair loss vary for their female counterparts. Skin disruption in animal models has induced follicular neogenesis by stimulating the dermis’ natural wound healing process (Ito et al., 2007, Kim et al., 2016). Powered skin disruption devices have shown clinical efficacy in men but have not yet been tested in a female population (Yu et al., 2018). In this pilot study, we investigate the use of scalp microwounding to promote hair growth in FPHL.

Eleven women with mild-to-moderate FPHL (Sinclair scores: 2–3.5) who had plateaued on stable FPHL treatment for ≥6 months were enrolled in this interventional pilot study. Patients were screened to exclude other hair loss disorders, as well as comorbidities that may affect hair growth or wound healing. Patient demographics are detailed in Table 1. Two women who were taking oral spironolactone for >1 year and who had plateaued on treatment continued for the duration of the trial.

**Table 1:** Patient demographics.

Characteristic	Value
**Age, y**	**n = 11**
Mean	49
Range	25–70
**Race, n (%)**	**n = 11**
Caucasian	9 (82)
Asian	1 (9)
African American	1 (9)
**Ethnicity, n (%)**	**n = 11**
Hispanic	2 (18)
Non-Hispanic	9 (82)
**Therapies failed/discontinued prior to microneedling, n (%)**	**n = 9**
Spironolactone	4 (36)
Topical minoxidil (restarted with microneedling)	5 (45)
Low-level laser light device	2 (18)
Finasteride	1 (9)
Plasma rich platelet injections	2 (18)
**Therapies continued during microneedling, n (%)**	**n = 4**
Spironolactone	2 (18)
Topical minoxidil	3 (27)
**Baseline Sinclair score, n (%)**	**n = 11**
2	1 (9)
2.5	4 (36)
3	5 (46)
3.5	1 (9)

Patients underwent six treatments over the course of 3 months with the Follica Hair Follicle Neogenesis Device (Follica, Boston, MA), a proprietary, powered microneedling device. Topical lidocaine cream was applied for 20 minutes prior to treatment to minimize discomfort and smooth hair. The treatment area included the frontal, crown, and vertex scalp and extended laterally to the upper-parietal scalp. The device was passed over the treatment area twice at each session, extending outward from the central part in the direction of hair strands to avoid tangling. Patients applied topical minoxidil 5% foam nightly to these areas for the duration of the trial. Topical minoxidil foam was not applied on treatment days. The study endpoints included global photographs, physician documented Sinclair score, and patient-reported improvement at 4 months.

Ten of 11 patients reported perceived improvement in hair growth at the end of the study. Six patients categorized their hair as slightly better, three patients as moderately better, and one patient as significantly better. All 11 patients demonstrated improvement in physician-graded Sinclair scores after 4 months, with changes ranging from 1 to 1.5. The average improvement in Sinclair grade was just above 1 full integer change (Fig. 1).

**Fig. 1:**
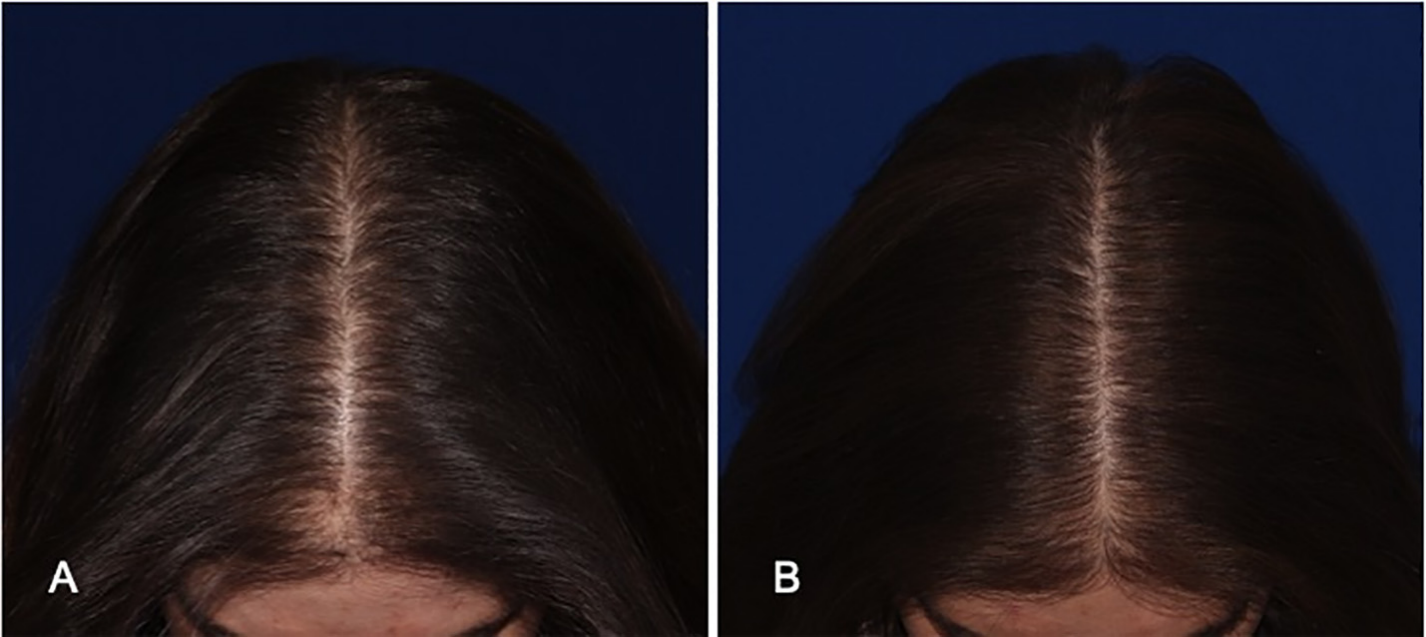
Female pattern hair loss (A) at baseline with Sinclair score 2.5 and (B) after six treatments with the Follica hair follicle neogenesis powered microneedling device, with Sinclair score 1.5.

Patients were asked to rate the pain of the procedure from 0 to 10, with higher scores indicating more discomfort. The average reported score was 6.4, which was limited to the procedure time. Other adverse events included transient headache, pinpoint bleeding, and mild erythema that resolved within 24 hours. There were no incidences of infection.

Skin disruption with the Follica Hair Follicle Neogenesis Device improved physician-reported Sinclair scores by at least 1 integer over the course of 4 months. All but one patient reported self-perceived improvement in hair coverage when comparing baseline and end-of-study global scalp photographs. This represents both rapid growth and improvement that was substantial enough to be appreciated on a global scale by both physician and patient. The procedure is reportedly uncomfortable but tolerable, and all patients enrolled completed the series of treatments. Adverse events reported were mild and self-resolving.

Scalp skin disruption demonstrated efficacy in this population of treatment-recalcitrant as well as in treatment-naïve women. Although limited by sample size, the results of this pilot study are encouraging and warrant larger studies in female cohorts.

## Conflicts of interest

None.

## Funding

Devices and topical minoxidil provided by Follica, Inc.

## Study approval

The author(s) confirm that any aspect of the work covered in this manuscript that has involved human patients has been conducted with the ethical approval of all relevant bodies. Partners Healthcare institutional review board protocol # 2018P001981.
